# Enhanced electrical and magnetic properties of (Co, Yb) co-doped ZnO memristor for neuromorphic computing

**DOI:** 10.1039/d3ra06853f

**Published:** 2023-12-11

**Authors:** Noureddine Elboughdiri, Shahid Iqbal, Sherzod Abdullaev, Mohammed Aljohani, Akif Safeen, Khaled Althubeiti, Rajwali Khan

**Affiliations:** a Chemical Engineering Department, College of Engineering, University of Ha'il P.O. Box 2440 Ha'il 81441 Saudi Arabia; b Chemical Engineering Process Department, National School of Engineers Gabes, University of Gabes Gabes 6029 Tunisia; c Department of Physics, University of Wisconsin La Crosse WI USA; d Engineering School, Central Asian University Tashkent Uzbekistan; e Scientific and Innovation Department, Tashkent State Pedagogical University Named After Nizami Tashkent Uzbekistan; f Department of Physics, University of Poonch Rawalakot Rawalakot 12350 Pakistan; g Department of Chemistry, College of Science, Taif University P.O. BOX. 110 21944 Taif Saudi Arabia; h Department of Physics, University of Lakki Marwat Lakki Marwat 2842 KP Pakistan rajwali@ulm.edu.pk khan_phy@foxmail.com; i Department of Physics, United Arab Emirates University United Arab Emirates

## Abstract

We investigate the morphological, electrical, magnetic, and resistive switching properties of (Co, Yb) co-ZnO for neuromorphic computing. By using hydrothermal synthesized nanoparticles and their corresponding sputtering target, we introduce Co and Yb into the ZnO structure, leading to increased oxygen vacancies and grain volume, indicating grain growth. This growth reduces grain boundaries, enhancing electrical conductivity and room-temperature ferromagnetism in Co and Yb-doped ZnO nanoparticles. We present a sputter-grown memristor with a (Co, Yb) co-ZnO layer between Au electrodes. Characterization confirms the ZnO layer's presence and 100 nm-thick Au electrodes. The memristor exhibits repeatable analog resistance switching, allowing manipulation of conductance between low and high resistance states. Statistical endurance tests show stable resistive switching with minimal dispersion over 100 pulse cycles at room temperature. Retention properties of the current states are maintained for up to 1000 seconds, demonstrating excellent thermal stability. A physical model explains the switching mechanism, involving Au ion migration during “set” and filament disruption during “reset.” Current–voltage curves suggest space-charge limited current, emphasizing conductive filament formation. All these results shows good electronic devices and systems towards neuromorphic computing.

## Abstract 2

Functional comparison between a biological synapse and a memristor.

## Graphical abstract

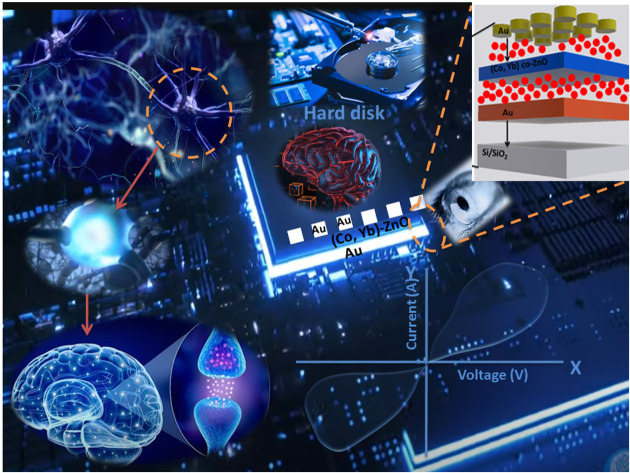

## 1. Introduction

**Fig. 1:**
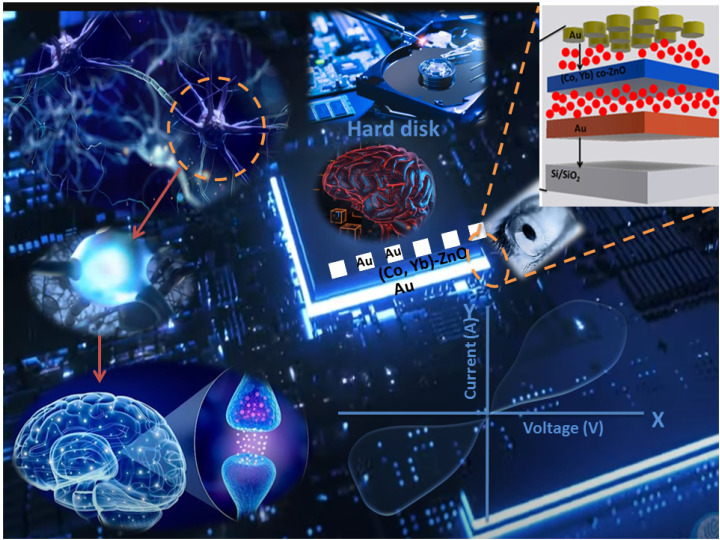
Functional comparison between a biological synapse and a memristor. A biological synapse and its junction. A memristor structured as Au/(Co,Yb)co-doped ZnO/Au representing an electronic synapse and their corresponding applications.

Neuromorphic computing is a cutting-edge field that seeks to emulate the remarkable computational capabilities of the human brain through the development of brain-inspired hardware and algorithms.^1,2^ At the heart of this endeavor lies the quest for artificial synapses that can replicate the dynamic nature of biological neural connections.^3–5^ Memristors, a class of novel electronic devices, have emerged as a pivotal component in this pursuit, holding the potential to revolutionize the landscape of computing.^6,7^ This introduction delves into the significance of memristors in neuromorphic computing, elucidating their role in synaptic emulation, the intricacies of pre-synaptic and post-synaptic functions, their unique *I*–*V* characteristics, and their diverse applications in this transformative field.^8^ Memristors are hailed as the electronic analogs of synapses due to their capacity to mimic this plasticity.^9^ They can modify their electrical resistance in response to the voltage applied, which enables them to replicate the weight adjustments of biological synapses during learning and memory processes. Central to memristor operation in neuromorphic computing are their unique current–voltage (*I*–*V*) characteristics. Memristors exhibit non-linear *I*–*V* curves, often described as pinched hysteresis loops.^10^ This peculiarity allows them to store and retrieve information in a manner analogous to the variable strength of biological synapses. The gradual resistance changes within a memristor, influenced by applied voltage pulses, facilitates the mimicking of synaptic plasticity, which is essential for learning, adapting to new information, and memory formation.^7,11^ The integration of memristors in neuromorphic computing systems brings forth a multitude of applications. These include memristor-based neuromorphic computing accelerating AI tasks such as pattern recognition, energy deficiency, latency, constraint budget, speech processing, and natural language understanding by emulating the brain's parallel processing and synaptic plasticity.^12,13^ The complete description is shown in Fig. 1.

One particularly intriguing avenue of exploration within this field is the incorporation of ferromagnetic zinc oxide (ZnO) nanoparticles.^14,15^ Ferromagnetic ZnO nanoparticles have emerged as a promising candidate due to their unique combination of magnetic and semiconductor properties, offering the potential to revolutionize the field of neuromorphic computing.^16^ In this introduction, we explore into the significance of ferromagnetic ZnO nanoparticles in the context of neuromorphic computing, explain their properties, discuss their potential applications, and provide insights into ongoing research. However, recent advancements in materials science have unveiled an exciting aspect of ZnO: its ferromagnetic behavior.^17^ This unexpected discovery has opened up new prospects for exploiting ZnO in a multitude of electronic and magnetic applications. Ferromagnetic ZnO nanoparticles, with their small size and unique magnetic properties, have gained significant attention due to their potential to bridge the gap between conventional electronics and brain-inspired computing.^18^

In the context of enhancing the electrical and magnetic properties of (Co, Yb) co-doped ZnO memristors for neuromorphic computing, the systematic doping of ferromagnetic dopants such as cobalt (Co), nickel (Ni), ytterbium (Yb), gadolinium (Gd), and titanium (Ti) into ZnO nanoparticles has emerged as a critical area for advancing cognitive computing capabilities.^19,20^ This kind of doping approach enables the manipulation of material properties to achieve specific functionalities crucial for neuromorphic computing applications. Co doping within ZnO nanoparticles has garnered substantial interest due to its ability to introduce ferromagnetism into the semiconductor matrix.^21,22^ Pioneering work by Djerdj *et al.* has demonstrated the successful incorporation of Co ions into ZnO nanoparticles, thereby inducing ferromagnetic behavior at room temperature.^23^ This achievement has paved the way for precise control over magnetic impurities, thereby enabling tunable synaptic behavior and memory functions within neuromorphic systems. Similarly, nickel (Ni) doping has been explored to enhance the ferromagnetic properties of ZnO nanoparticles. Wang *et al.* reported significant improvements in ferromagnetic behavior in Ni-doped ZnO nanoparticles compared to their undoped counterparts.^24^ This enhancement holds great promise for optimizing information programming and retrieval processes within neuromorphic systems. Gadolinium (Gd) doping in ZnO nanoparticles offers the ability to modify the magnetic properties of the material. Mazhdi *et al.* have highlighted how Gd doping allows for the fine-tuning of ferromagnetic behavior, which proves highly advantageous in achieving precise control over synaptic plasticity within the field of neuromorphic computing.^25^ Moreover, the introduction of titanium (Ti) doping represents another noteworthy advancement, giving ZnO nanoparticles with a dual functionality. The pervious researchers have showcased that Ti-doped ZnO nanoparticles exhibit both ferromagnetic and photoluminescent behavior, opening up exciting possibilities for combining magnetic and optical information processing in neuromorphic computing, thus adding a new dimension to cognitive computing.^26^ The incorporation of ferromagnetic dopants like Co, Ni, Yb, Gd, and Ti into ZnO nanoparticles represents a promising path forward in the realm of neuromorphic computing.^20^ These materials offer a unique combination of magnetic and semiconductor properties, setting the ground for the development of artificial synapses with highly tunable functionalities. As research in this field advances, the convergence of ferromagnetic doping and neuromorphic computing holds the potential to explore cognitive computing capabilities, ultimately accompanying in intelligent technology that can match human-like thinking, learning, and adaptability. In this study, we employ a hydrothermal technique to synthesize both undoped and (Co, Yb) co-doped ZnO nanoparticles. We thoroughly investigate changes in the structural and magnetic characteristics of (Co, Yb) co-doped ZnO, focusing on the dependence of nanoparticle size after doping. Additionally, we investigate the role of oxygen vacancies concerning the heat applied during the doping process, explaining how temperature fluctuations impact crystallinity and defect density. Notably, the incomplete oxidation of Zn^2+^ to Zn^4+^ during higher doping contributes to the improved crystallinity and defect density when the temperature decreases. To experimentally validate our findings, we fabricate a sputter-grown memristor with a (Co, Yb) co-doped ZnO layer sandwiched between 100 nm-thick Au electrodes. Characterization studies confirm the presence of the ZnO layer and the integrity of the Au electrodes. Our memristor exhibits repeatable equivalent resistance switching capabilities, allowing for precise manipulation of conductance between low and high resistance states. Statistical strength tests further confirm the stability of resistive switching, demonstrating minimal dispersion over 120 pulse cycles at room temperature. Moreover, retention properties exhibit the remarkable conservation of four distinct current states for up to 10 000 seconds, highlighting the excellent thermal stability of our designed memristor. Through these investigations, we contribute to the area of neuromorphic computing by advancing our understanding of (Co, Yb) co-doped ZnO materials and their potential in realizing high-performance memristive devices, setting the ground for the development of more efficient and adaptive cognitive computing systems.

## 2. Experimental procedure

### 2.1 Materials

Zinc acetate [Zn(CH_3_COO)_2_·H_2_O], cobalt acetate [Co(CH_3_–COO)_2_·4H_2_O], and ytterbium acetate [Yb(CH_3_COO)_2_·4H_2_O] were procured from Alfa-Aesar. All reagents used in the experiments were employed as received without the need for additional purification.

### 2.2 Nanoparticles synthesis and device fabrication

**Fig. 2:**
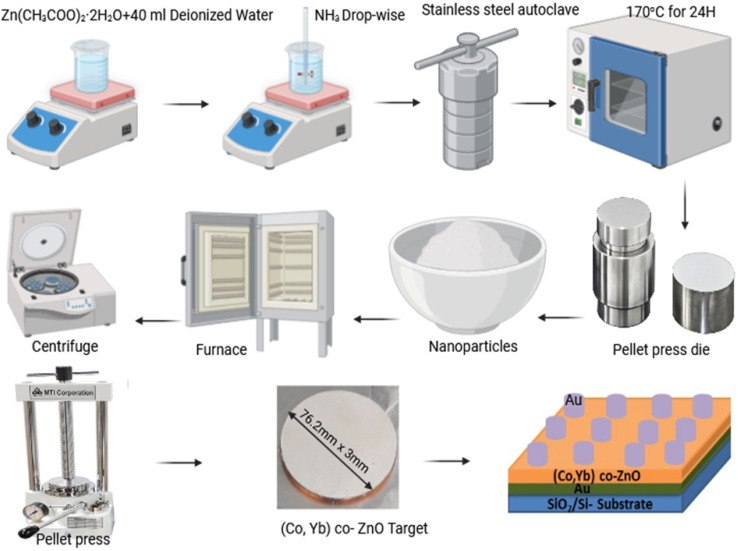
The schematic of the hydrothermal synthesis process of (Co, Yb) co-ZnO NPs and Au/3% (Co,Yb) co-ZnO/Au/Si–SiO_2_ device, from co-doped ZnO powder to device.

The synthesis of both undoped and (Co, Yb) co-doped ZnO samples was carried out using the hydrothermal method, as shown in Fig. 2. In brief, zinc acetate [Zn(CH_3_COO)_2_·H_2_O] was dissolved in 40 ml of deionized water, followed by the slow addition of 26 ml of aqueous ammonia solution (2 M) under vigorous stirring, maintaining a pH range between 1.0 and 10.2.^27,28^ Once the reaction was completed, the solutions were transferred into a stainless steel autoclave and placed in an oven at 170 °C for 24 h. Afterward, white precipitates were collected *via* centrifugation and washed with distilled water. These washed precipitates were then dried at 50 °C for 24 h with increasing 1°C min^−1^ in an electric furnace. Finally, the resulting ZnO nanopowder was annealed at 500 °C for 4 h using an automated electric furnace, as shown in the detailed schematic diagram of Fig. 2. In the case of Co and Yb co-doped samples, Zn (CH_3_COO)_2_·H_2_O, Co (CH_3_–COO)_2_·4H_2_O, or Yb (CH_3_COO)_2_·4H_2_O was gradually added to the former solution, leading to the formation of precipitates that were further stirred for 40 min.

Memristive systems comprising Au/3% (Co,Yb) co-ZnO/Au/Si–SiO_2_ were devised. Initially, (Co,Yb) co-ZnO nanoparticles were introduced into a pellet press die with a 7.62 mm diameter and compressed using a hydraulic pellet presser. Subsequently, a pellet measuring 7.62 mm in diameter and 3 mm in thickness was affixed to a Cu target (7.62 mm diameter, 3 mm thickness) using indium glue, thus forming the appropriately shaped target for sputtering fabrication. The (Co,Yb) co-ZnO material was then deposited onto a pre-existing Au/Si–SiO_2_ substrate, as shown in the Fig. 2. Finally, a top electrode composed of Au, 100 nm in thickness, was deposited *via* DC magnetron sputtering employing a stoichiometric Au target with 99.999% purity (AEM Ltd. China). A shadow mask was employed to deposited electrode patterns with an area of 10 μm^2^. Sputtering of the Au target was conducted using an Ar gas flow rate of 35 sccm, resulting in a total working pressure of 5 × 10^−4^ mBar. A current of 150 mA (power ∼45 W) was applied to the Au sputtering target, resulting in a consistent deposition rate of 1 Å s^−1^. During deposition, the samples were rotated at 10 rpm to ensure uniform film formation. A detailed illustration of the device febrication is provided in Fig. 2. All the room-temperature *I*–*V* characteristics were achieved with a source meter system (Keithley 2612B).

### 2.3 Experimental tools used for nanoparticles

Structural characterization was conducted using X-ray diffraction (XRD) with Cu Kα radiation (*λ* = 1.5406 Å). Lattice parameters and their corresponding volumes were determined using standard Rietveld refinements through High Score Plus. Chemical composition analysis was performed utilizing energy-dispersive X-ray (EDX) spectroscopy integrated into the field emission scanning electron microscope (FE-SEM). In order to determine the lattice parameters, such as the size (volume) and spacing between atoms (*d*-spacing) brand-new HighScore Plus software was employed to perform Rietveld refinements. The particles' sizes were measured using a field transmission electron microscope (TEM). X-ray photoelectron spectroscopy (XPS) measurements were carried out using a Thermo ESCALAB 250 with a monochromatic Al-Kα X-ray source (*hν* = 1486.6 eV). Dielectric properties were assessed using Agilent Impedance Analyzer and LCR meters within a frequency range of 40 Hz–5 MHz. Magnetic properties were measured using a Quantum Design superconducting Quantum Interface Device (SQUID) Magnetic properties measurement system.

## 3. Results and discussion

### 3.1 Structural analysis

**Fig. 3:**
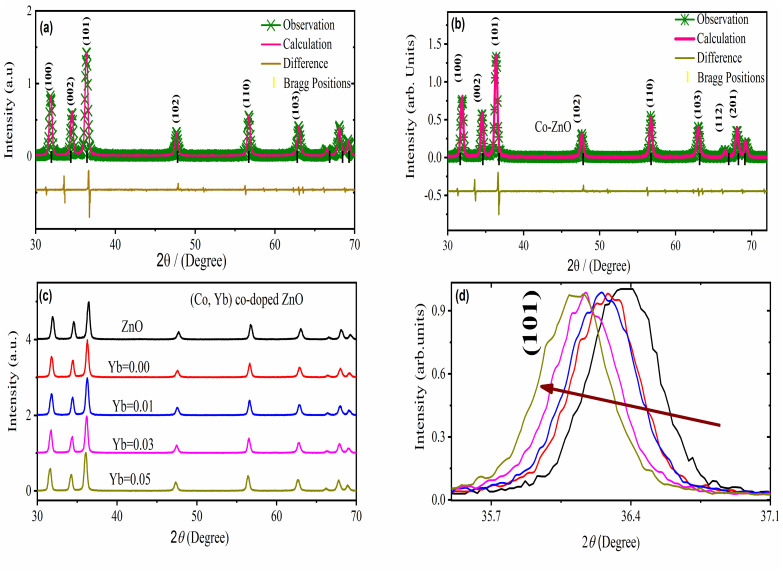
The Rietveld-refined X-ray diffraction pattern of (a) ZnO, and (b) Co–ZnO. (c) XRD pattern of all the (Co, Yb) co-ZnO nanoparticles. (d) The comparison X-ray diffraction patterns peaks of the Co-doped ZnO and for (Co, Yb) co-ZnO nanoparticles.

Fig. 3 illustrates the X-ray diffraction (XRD) patterns of both undoped and (Co, Yb) co-doped ZnO samples. These patterns reveal that all the samples are single-phase, devoid of impurities, and possess a crystalline structure with a hexagonal lattice (wurtzite-type *P*6_3_*mc*) exhibiting a preferred orientation along the (101) plane (Fig. 3(b and c). Notably, there are no additional peaks observed in the spectra following the (Co, Yb) co-doping process, indicating the formation of a solid solution across all the concentrations of (Co, Yb) utilized in this study. It was observed that the XRD peaks shift to lower angle with Yb doping (as shown in Fig. 3(b)) because the substitution of smaller Zn ions with larger Yb ions in the ZnO lattice leads to local lattice expansion. All the lattice constants of *a* = 3.2402 Å, *c* = 5.1870 Å and *a* = 3.2549 Å, *c* = 5.1999 Å were acquired by Rietveld refinement software along with weighted profile factor *R*_WP_ = 9.36%, 9.36 and the goodness-of-fit *χ*^2^ = 2.574, 2.578) for the pure ZnO and Zn_0.96_Co_0.05_Yb_0.05_O as shown in Fig. 3(a). This expansion increases the interatomic distances, resulting in a decrease in the Bragg angle and a corresponding shift of XRD peaks to lower angles, as shown in Fig. 3(b). This shift is a characteristic signature of lattice parameter changes induced by dopants in the crystal lattice. The ZnO crystal structure was unaffected by the 5-percent Co-doping into the parent molecule. The lattice parameters of all the pure, Co–ZnO and (Co, Yb) co-ZnO samples were calculated with GSAS. The lattice constants *a* and *c* are easily observable and are growing steadily larger with increasing doping levels (Fig. 3(c)), indicating that Co^2+^ and Yb^3+^ may be used to replace Zn^2+^ atoms throughout the Co–ZnO and (Co, Yb) co-ZnO structure. This makes sense given that Co^2+^ ions have smaller radii (0.058 nm) and lower valence states (compared to the Zn^2+^ ions (0.060 nm) they are hosting) than Co^2+^ ions themselves but lower than the Yb^3+^ (0.100 nm). Peak intensity and crystallization also improve at higher doping concentrations, while defects get smaller. In addition, all pure and doped-ZnO nanoparticles have larger lattice parameters than bulk ZnO. It stands to reason, as raising the doping level helps grains expand. Table 1 lists several material properties such as lattice constants, volume, grain size, *etc.* This fluctuation in ionicity of the injected Co and Yb into ZnO or the possible generation of oxygen vacancies in the crystal lattice have been linked to the observed rise in lattice parameter for ZnO nanoparticles,^22^ which is consistent with prior studies. According to our findings, the structural properties of ZnO samples are strongly influenced by particle size. Increasing Yb substitution causes a broadening of Co-doped ZnO exhibits broadening of its XRD peaks and an expand their FWHM (full width at half maximum),^23^ demonstrating that ZnO lattice distortion occurs as a result of the various dopant ions (related to ionic radius). Analyzing the XRD peak widths using the Scherrer formula,^29^

1 *d* = 0.9*λ*/*β* cos *θ*

where *d* represents the particle's size, the glancing angle (2/2) and the FWHM (full width at half maximum). It is clear that the average crystalline diameters of all the annealed (ZnO–Ni) samples fall within the range of 21.2–26.3.

**Table 1:** XRD-estimated calculated dimension of structure values nanoparticles comprised of pure ZnO and (Co, Yb) co-ZnO

Sample	*hkl*	*d*-spacing (Å)	2*θ* (°)	Grain size (nm)	Lattice constant		*c*/*a*	*u*	Unite cell volume (Å^3^)
					*a* (Å)	*c* (Å)			
ZnO	100	2.7973	31.832	14.34	3.2420	5.180	1.596	0.388	47.45
	002	2.5972	34.423	13.11	—	—			—
	101	2.4704	36.344	11.45	—	—			—
Zn_0.96_Co_0.05_O	100	2.8079	31.847	13.54	3.2442	5.1950	1.599	0.392	47.40
	002	2.5973	34.645	13.61	—	—			—
	101	2.4715	36.465	12.34	—	—			—
Zn_0.94_Co_0.05_Yb_0.01_O	100	2.8082	31.902	12.32	3.2446	5.1980	1.599	0.395	47.37
	002	2.5976	34.670	13.35	—	—			—
	101	2.4719	36.490	14.01	—	—			—
Zn_0.92_Co_0.05_Yb_0.03_O	100	2.8085	36.498	14.75	3.2515	5.1990	1.602	0.399	47.28
	002	2.5980	34.689	15.54	—	—			—
	101	2.4721	36.512	16.4	—	—			—
Zn_0.90_Co_0.05_Yb_0.05_O	100	2.8088	36.459		3.2543	5.1999	1.605	0.401	47.21
	002	2.5984	34.692						

The XRD peaks broadening were used for the Scherrer formula to determine the crystallite size of specimens,^30,31^ and it was found that size decreased with an increase in Co content from 0 to 5%. Such a decrease can be attributed to grain growth inhibition caused by the presence of Co and Yb in ZnO. This shows that the presence Co in high amounts does not only provide O_2_ vacancies to smooth densification to support and inhibits grain development. However, grain boundary segregation in highly doped specimens causes a reduction in particle size. The increase in lattice parameter with a higher in Co and Yb content is because of the higher ionic radius of Co (70 pm) and Yb (100 pm) when compared to Zn (74 pm) ions. Table 1 summarizes the calculated X-ray density, grains size, lattice parameters, grain size, and *V*.

**Fig. 4:**
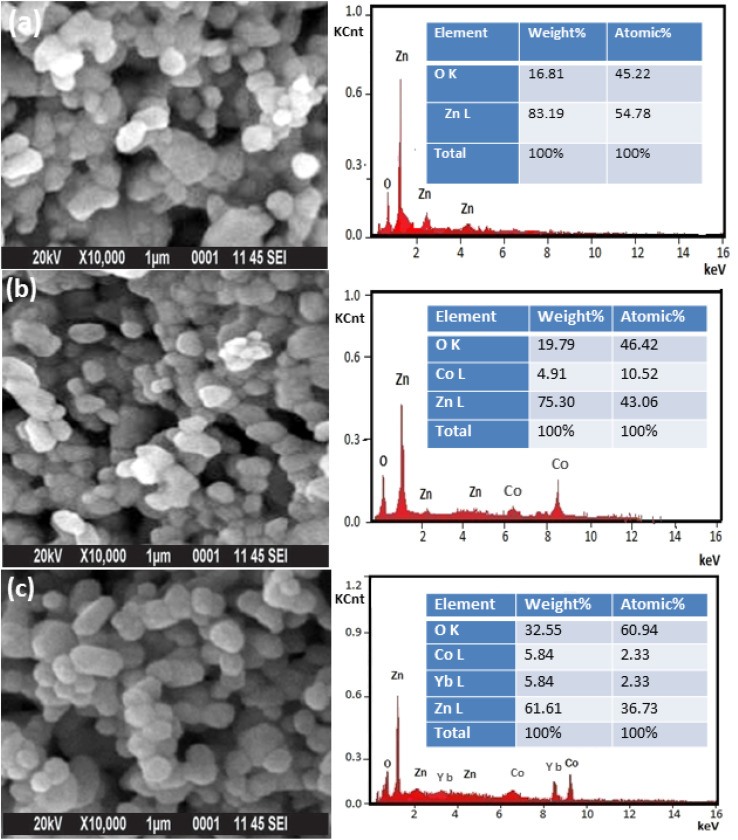
(a–c) shows the SEM image and their corresponding (right panel of Fig. 2) EDX of (a) ZnO, (b) Zn_0.95_Co_0.05_O and (c) Zn_0.92_Co_0.05_Yb_0.05_O NPs.

Fig. 4(a–c) displays the morphology and elemental analysis of pure, 5 wt% Co and (Co, Yb) co-doped ZnO examined by SEM analysis. These data imply that the NPs have varied shapes and a wurtzite structure with hexagonal symmetry. The shape of the samples transforms from a rectangle to a structure resembling a sphere as the Co and Yb content increases. The EDX spectra depicted in Fig. 4(a–c) provide a comprehensive insight into the elemental compositions, given in weight percentages (wt%), of Zn, O, Co, and Yb. In line with expectations, pure ZnO predominantly consists of Zn and O elements. However, in the case of Co-doped and Yb co-doped ZnO specimens, discernible peaks for Co and Yb are observed, validating the successful introduction of these dopants into the material. Notably, the examination of the EDX results reveals that the weight percentages of the doped transition metals closely align with the prescribed quantities used during the sample preparation process, as evidenced in the right panel of Fig. 4(a–c). This alignment underscores the accuracy of the doping process and the precision in achieving the desired composition. Furthermore, SEM analysis, as illustrated in Fig. 4(a–c), provides a detailed assessment of the morphology and elemental composition of pure ZnO, 5 wt% Co-doped ZnO, and 5 wt% Yb co-doped ZnO. These findings indicate that the nanoparticles in all cases maintain a consistent morphology characterized by a hexagonal wurtzite structure. Additionally, it is worth noting that with an increase in the Co and Yb content, the samples exhibit an irregular-like structure with an elevated degree of aggregation. This change in morphology can be attributed to the influence of the dopant elements, Co and Yb, on the growth and arrangement of the nanoparticles, which results in this distinctive structure.

**Fig. 5:**
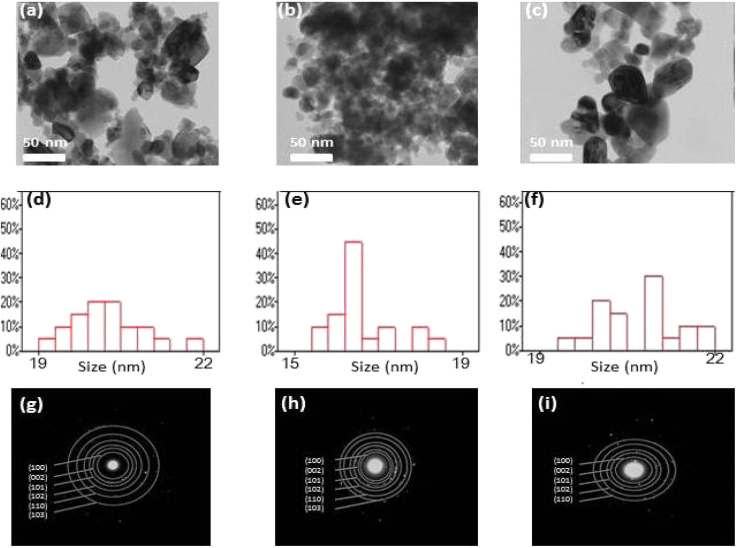
The TEM images of the sample, (a) ZnO, (b) 5% Co–ZnO and (c) 5% (Co, Yb) co-ZnO NPs. (d–f) shows the particle size distribution of ZnO, 5% Co–ZnO and 5% (Co, Yb) co-ZnO NPs. (g–i) shows the selected area electron diffraction (SEAD) images.

Fig. 5(a–i), shows the transmission electron microscopy (TEM) images of ZnO, Co–ZnO and Zn_0.95_Co_0.05_Yb_0.05_O NPs. The TEM images show almost spherical-shaped particles with a uniform distribution for both the un-doped, Co-doped ZnO and Zn_0.92_Co_0.05_Yb_0.05_O samples. The particle size distribution is quite narrow, and all of the particles have a form reminiscent to primatology. It is determined that the size of the crystallites is between 16 and 22 nm. In addition, the rapid nucleus production of the crystal may be aided by nuclear aggregation at higher doping. To a large extent, the product's structure and morphology are determined by the pace at which particles aggregate. Likewise, in Fig. 5(c) and (f), the particle size distribution of (Co, Yb) co-doped ZnO is depicted, specifically highlighting the impact of Co doping at a concentration of 5%. The particle sizes in this case range from 15.87 to 22.53 nm, with a mean particle size of 91.26 ± 1.12 nm. Notably, as the Co concentration increases, there is a notable reduction in particle size. This phenomenon can be attributed to the substitution of Co^2+^ and Yb ions for Zn^2+^ within the lattice structure. Co^2+^ ions have a smaller ionic radius of 0.06 nm compared to Zn^2+^ ions, which have an ionic radius of 0.065 nm. This substitution causes the lattice to contract due to the smaller size of Co^2+^ ions, consequently leading to a decrease in particle size with increasing Co^2+^ concentrations. On the other hand, the larger ionic radius of Yb ions (0.1 nm) also contributes to this effect, further influencing the reduction in particle size.

Fig. 5(g–i) displays selected area electron diffraction (SEAD) images of the synthesized NPs. The fringes achieved in the SAED image for the ZnO sample indicate the formation of the polycrystalline tetragonal structure of ZnO. It is also essential to note that the broad XRD peak of Co and Yb co-doped ZnO suggests small crystallite size and, consequently, a significant surface area. It is evident from the TEM micrograph that the size of Co–Yb co-doped ZnO is greater than that of pure ZnO, implying that the surface area of Co–Yb co-doped ZnO is greater than that of pure ZnO. A larger surface area of the sample is required for enhanced photodegradation efficiency.

**Fig. 6:**
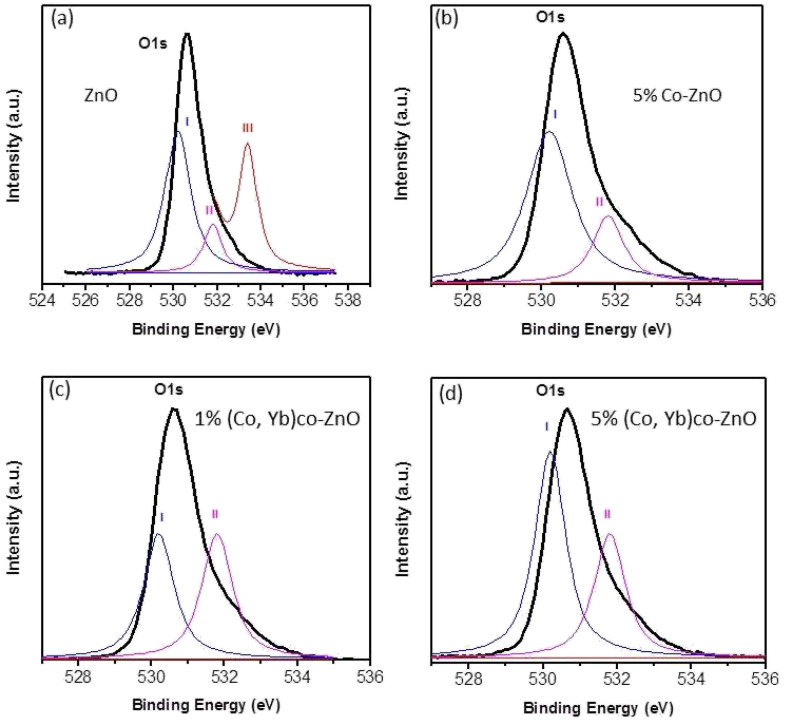
O 1s XP spectra of (a) ZnO nanoparticles (b) Zn_0.95_Co_0.05_O nanoparticles (c) Zn_0.94_Co_0.05_Yb_0.01_O nanoparticles, and (d) Zn_0.94_Co_0.05_Yb_0.05_O NPs.

To conduct a comprehensive examination of oxygen vacancies in ZnO, Zn_0.95_Co_0.05_O, Zn_0.94_Co_0.05_Yb_0.01_O, and Zn_0.94_Co_0.05_Yb_0.05_O NPs, we employed X-ray Photoelectron Spectroscopy (XPS) to analyze the O1s spectra, as presented in Fig. 6(a–d). These spectra for both pure ZnO, 5% Co–ZnO nanoparticles and co-doped ZnO with Co and Yb can be de-convoluted into three Gaussian-distributed peaks centered at 530.2 eV, 531.8 eV, and 533.4 eV, respectively.^32,33^ Specifically, Fig. 6(a–d) showcases the O1s peak at 533.4 eV. Peak III, located at 533.4 eV, is a consequence of the presence of loosely bound oxygen on the ZnO surface. Importantly, this peak consistently resides outside the Gaussian distribution for the O 1s signal across all samples, signifying the absence of loosely bound oxygen on the surfaces of all ZnO, Zn_0.95_Co_0.05_O, Zn_0.94_Co_0.05_Yb_0.01_O, and Zn_0.94_Co_0.05_Yb_0.05_O nanopowders. The lower binding energy component at 530.2 eV (referred to as Peak I) corresponds to O^2−^ ions within the hexagonal wurtzite structure of Zn^2+^ ions.^34^ The middle peak centered at 531.8 eV (indicated as Peak II) is attributed to O^2−^ ions within oxygen-deficient regions in the ZnO matrix.^33^ The intensity of this peak varies in response to fluctuations in the concentration of oxygen vacancies, with an increased intensity indicating a greater presence of oxygen vacancies (*i.e.*, oxygen-deficient regions) in ZnO. Notably, the intensity of this peak amplifies with an increase in the concentration of dopants, reaching its highest level in the case of Zn_0.94_Co_0.05_Yb_0.01_O nanoparticles, as shown in Fig. 6(d). This substantiates the notion that augmenting the dopant concentration (Co and Yb) within ZnO results in an increase in oxygen vacancies. It is noteworthy that the XPS findings align closely with the results obtained from X-ray Diffraction (XRD) and Energy-Dispersive X-ray Spectroscopy (EDX), further reinforcing our observations.

**Fig. 7:**
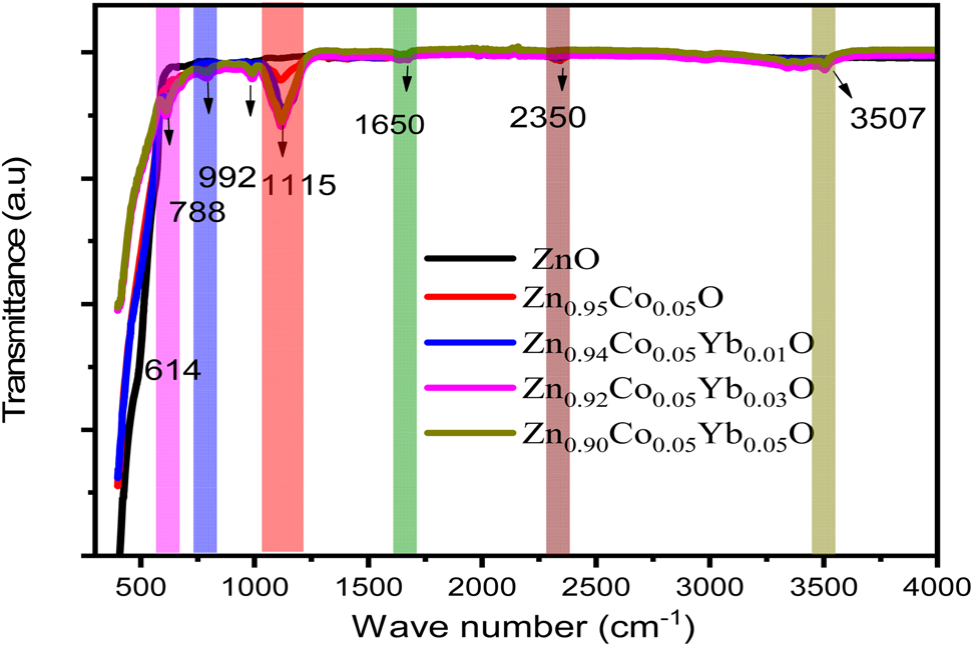
The FT-IR pattern of ZnO, Zn_0.95_Co_0.05_O, Zn_0.94_Co_0.05_Yb_0.01_O, Zn_0.92_Co_0.05_Yb_0.03_O and Zn_0.94_Co_0.05_Yb_0.05_O NPs.

Fig. 7(a) depicts the pure FT-IR, the ZnO, Zn_0.95_Co_0.05_O, Zn_0.94_Co_0.05_Yb_0.01_O, Zn_0.92_Co_0.05_Yb_0.03_O and Zn_0.94_Co_0.05_Yb_0.05_O NPs. All samples had predominant absorption bands at 3507, 1650, 1115, 995, 788, and 614 cm^−1^. The absorption spectra at 614 cm^−1^ corresponds to the Zn–O range. The absorbent band at 3507 cm^−1^ corresponds to the OH vibration of water. The band around 1650 cm^−1^ is caused by C=O stretching. Band of absorption detected at 2350 cm^−1^ resembles CO_2_ molecules in the air. In contrast, the absorption band at around 995 cm^−1^ indicates the shoulder of resonance interaction between modes of pulsation of oxide ions in nano-crystals with asymmetric stretching.

The bands that occur at around 788 cm^−1^ and 1115 cm^−1^ are due to the stretching of Zn–O–Zn and C–O. The band at 3507 cm^−1^ in the FTIR spectrum of the Co-doped ZnO sample corresponds to the –OH mode in H_2_O molecules. The presence of these manufactured NPs can be attributed to the atmospheric absorption of water molecules. The 995 cm^−1^ band consists of a shoulder with unequal stretching resonance contact between the vibrational modes of oxide ions in nano-crystals. The ZnO surface quickly absorbs the acetate and hydroxyl assemblies by drying them at temperatures exceeding 690 °C, consistent with the XRD output result, the FTIR result demonstrates that Co occupies the Zn site in the ZnO grid, as the zero peak in the spectrum is identical to that of Co.

**Fig. 8:**
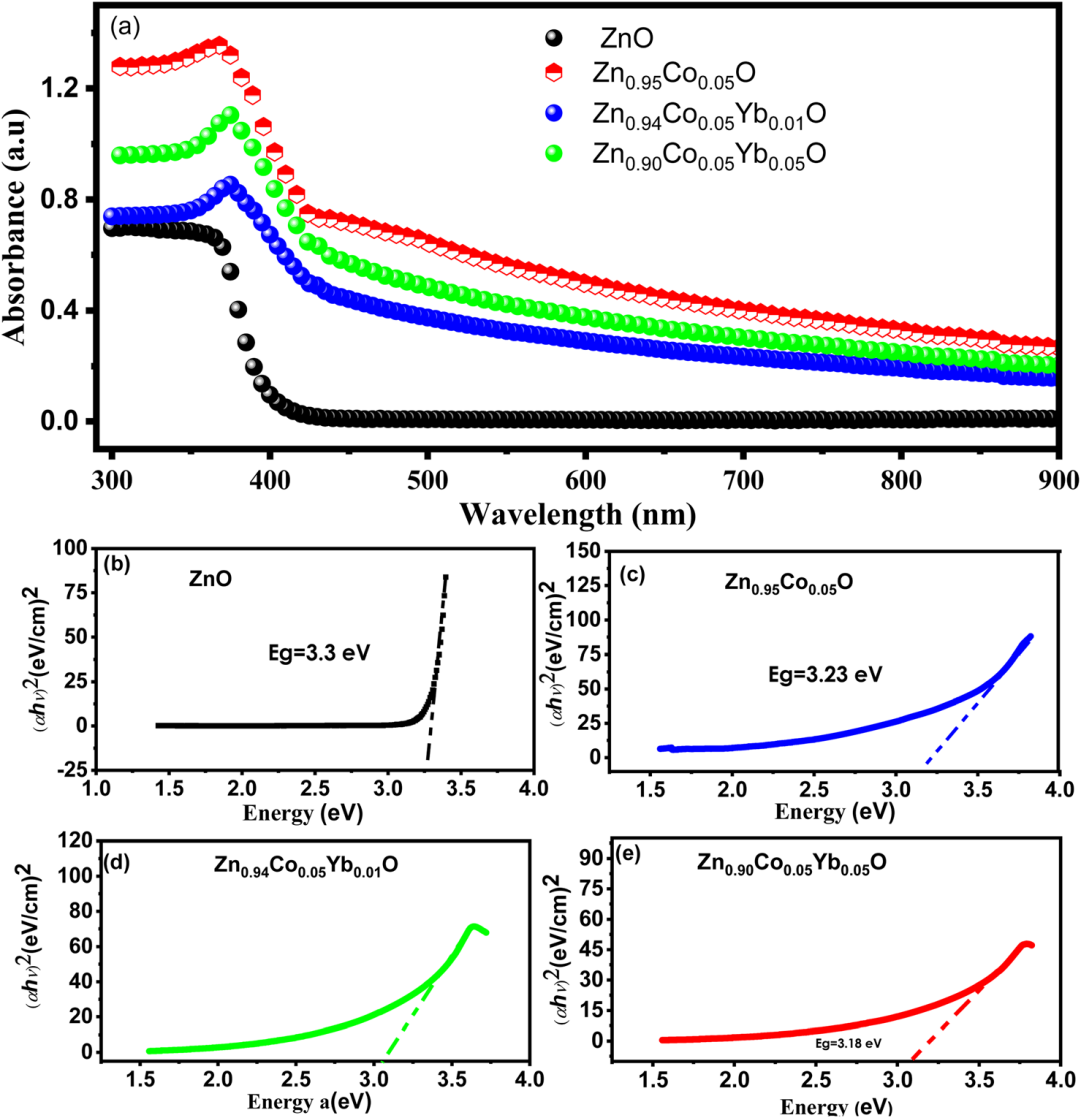
(a) UV-Vis spectroscopy ZnO, and Zn_0.95−*x*_Co_0.05_Yb_*x*_O (Co = 0.01 and 0.05) NPs. (b–e) calculation of energy band gap using Tauc plot method for all samples of ZnO, and Zn_0.95–*x*_Co_0.05_Yb_*x*_O (Co = 0.01 and 0.05).

We conducted an investigation into the optical properties of all samples using UV-vis spectroscopy, as illustrated in Fig. 8(a–e). In Fig. 8(a), the UV-vis absorption spectra are presented for both ZnO and Zn_0.95−*x*_Co_0.05_Yb_*x*_O (Co = 0.0, 0.01, and 0.04) NPs at room temperature, covering a wavelength range of 300–900 nm. Comparing these spectra to those of pure ZnO NTs, it becomes evident that Zn_0.95−*x*_Co_0.05_Yb_*x*_O (Yb = 0.0, 0.01, 0.05) NPs exhibit distinct and pronounced visible absorption peaks. Notably, as the dopant concentration increases, the absorption peaks of both ZnO and Zn_0.95−*x*_Co_0.05_Yb_*x*_O (Yb = 0.0, 0.01, 0.05) NPs shift towards longer wavelengths, specifically in the 370–410 nm range. This redshift in the absorption peaks implies that the size of the nanotubes increases with higher dopant concentrations. This increase in size can be attributed to the introduction of disorder and impurities into the ZnO crystal structure due to the presence of the dopant.^35–38^ Furthermore, electronic transitions of the dopant ions were analyzed across a wide spectral range (380–410 nm) using ellipsometry and optical analysis.^31,39^ These electronic transitions are induced by the presence of dopant ions, specifically Co^2+^ and Yb^3+^, which are situated in tetrahedral coordination within the crystal structure.^40–43^ Within the wurtzite structure of ZnO (*P*6_3_/*mmc* space group), tetrahedrally coordinated O^2−^ and Zn^2+^ ions naturally occur. The dopant ions Co^2+^ and Yb^3+^ replace host ions (Zn^2+^) in the absorption spectrum, leading to observable changes in the visible spectrum. Tauc relation was used to calculate the optical band gap (*E*_g_) of ZnO and Zn_0.95−*x*_Co_0.05_Yb_*x*_O (Co = 0.0, 0.01, 0.05).^44^

2 *αhυ* = *B*(*hυ* − *E*_g_)^*n*^

The coefficient of optical absorption (*α*) is represented by the equation *α* = *A*(*E* − *E*_g_)^*n*^, where *A* is a constant and *E* is the energy of the incident photon. In the case of ZnO, the value of *n* is ½, as it possesses a direct bandgap. Fig. 8(b–e) illustrate the extrapolated bandgap curves for both ZnO and Zn_0.95−*x*_Co_0.05_Yb_*x*_O (Yb = 0.0, 0.01, 0.05) NPs. Fig. 8(b) demonstrates that ZnO NPs have a bandgap of 3.43 eV, which is larger than that of the bulk material. Interestingly, as the doping concentration increases (with Co = 5% and Yb = 0%, 1%, and 5%), the bandgap steadily decreases, ranging from 3.45 eV to 3.15 eV, as depicted in Fig. 8(b–e).^45,46^ This reduction in the bandgap of ZnO NPs can be attributed to an exchange transition between the s–d and p–d electronic states, as explained by Bylsma *et al.*^47^ Furthermore, this decrease in the energy bandgap is a consequence of an increased surface-to-volume ratio and an elevated number of defects within the material.^45,46^ The observed redshift and reduction in the bandgap strongly indicate the incorporation of Co/Yb dopants into the ZnO lattice.^48^ The change in bandgap observed with Yb and Co co-doping in ZnO can be attributed to several interconnected factors. First, the introduction of Yb and Co ions into the ZnO lattice creates localized electronic states within the bandgap. These impurity states can interact with the host ZnO matrix, leading to the formation of intermediate energy levels within the bandgap. The hybridization of these impurity states with the host material alters the electronic structure of the composite, influencing its optical properties, including the bandgap.

Furthermore, the variation in bandgap can be linked to changes in the crystal structure and lattice parameters induced by the co-doping process. The incorporation of Yb and Co ions may result in lattice distortion and strain within the ZnO lattice. This distortion can impact the band structure and, consequently, the bandgap. The specific effect on the bandgap depends on the concentration and distribution of Yb and Co ions within the ZnO crystal lattice, as well as their electronic configurations. The observed redshift in optical properties is a multifaceted phenomenon rooted in several fundamental mechanisms. Quantum confinement, a consequence of reduced dimensions in nanostructures, results in discrete energy levels and can induce a redshift as the structure size increases. Changes in the crystal lattice, such as expansion or compression, can also lead to alterations in electronic band structures, contributing to redshift effects. Doping with specific atoms introduces localized energy levels, influencing absorption and emission spectra. Additionally, chemical interactions and electronic modifications stemming from dopants can impact the band structure. Quantum mechanical phenomena, defect-related transitions, and nanoparticle size and shape further contribute to the diverse array of mechanisms underlying observed redshifts. Understanding these mechanisms is pivotal for tailoring materials to desired optical characteristics.

### 3.2 Dielectric properties

#### 3.2.1 Dielectric constant

**Fig. 9:**
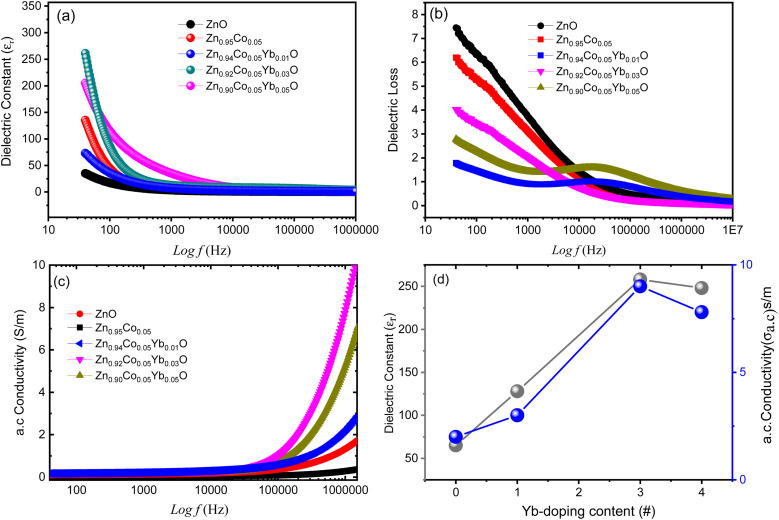
(a–c)The frequency dependence of the dielectric constant, dielectric loss and a.c electrical conductivity for ZnO and 0.1%,3% and 5% (Co, Yb) co-ZnO.(d) Dielectric constant and a.c conductivity *versus* Yb doping level.

Fig. 9(a) depicts the relationship between dielectric constant (*ε*_r_) and (Co, Yb) co-ZnO NPs at a specific calcination frequency (300 K) for a range of calcination temperatures. Capacitance measurements were taken using gold glue electrodes on pellets crushed to a diameter of 9 mm using a pressing machine to determine electrical conductivity, loss, and dielectric constant at 300 K for alternating current (AC). Khan *et al.*^25–29^ have previously utilized a technique with similar results. Using eqn (3), we were able to determine the *ε*_r_.

3 *ε*_r_ = *Cd*/*ε*_o_*A*

*ε*_r_ stands for the sample's relative dielectric permittivity (dielectric constant), *C* is the capacitance, *d* is the cylinder's height, and *A* is the pellet's cross sectional area. The dielectric permittivity of open space is denoted by *ε*_o_. The *ε*_r_ value of calcined is demonstrated that the mobility of nanoparticles of (Co, Yb) co-ZnO at doping concentration show a sharp reduction with rising *f* up to 1 × 10^4^ Hz, but then *ε*_r_ values at higher frequency remain nearly a constant. This decrease in dielectric constant at low frequencies can be attributed two different types of polarization, the two types of polarisation are space charge polarisation (SCP) and rotation dielectric polarisation (RDP), can explain this effect. There is a high concentration of defects (vacancies, *etc.*) at the interface. These O^2^ vacancies can be ionized either once or twice.^49^ In response to the application of an external field, a massive RDP is created by the alignment of a vacancies (holes) cause a big number of random dipole moments. Space charge polarization results when charges have a moment of inertia in opposition to the electric field and are held in place by a flaw in a nanostructure, which is caused by active grain boundaries and leads to significant polarizations and a large dielectric constant.^50^ Reversible dimerization and stoichiometry in (Co, Yb) co-ZnO nanoparticles are caused by their high specific surface area, which is in turn caused by the high surface to volume ratio. Fig. 9(a) shows that when the concentration of the dopant (Yb) increases, the dielectric constant decreases, demonstrating the strong size and doping dependence of dielectric characteristics. More structural disorder is introduced into the ZnO matrix when Co^2+^ and Yb^3+^ atoms with bigger radii are incorporated into the material,^51^ causing *ε*_r_ to drop.

#### 3.2.2 Dielectric loss

Fig. 9(b) shows the frequency dependence of the imaginary part of dielectric loss (*ε*′′) of (Co, Yb) co-ZnO that has been doped with different Yb content. If we raise the doping content the frequencies at which the relaxation peaks appear move up. Whenever the field frequency is exactly equal to the hopping peak relaxation times correspond to charge-carrier frequency ranges are detected. This behavior is because of the defect dipoles are responsible for the upshifting in relaxation peaks shifting upwards in frequency. SCP is to blame for the increased dielectric loss at low frequencies. The provides a mechanistic explanation for this phenomenon.^50^ This mechanism postulates that at low and intermediate fs(Hz), SCP is caused by bulk impurity ions (dopants) capturing the surface electron. Fig. 9(b) shows that *ε*′′ rises from very low to very high fs(Hz), peaks at about middle fs(Hz), and then gradually decreases as *f*(Hz) climbs tan *δ* becomes smaller as calcination temperature increases can be attributed to the fact that larger particles have a lower fault density.

#### 3.2.3 Electrical conductivity

Fig. 9(c) shows the frequency dependence of the imaginary part of electrical conductivity (*α*_a.c._) of (Co, Yb) co-ZnO that has been doped with different Yb content. Another, as shown in Fig. 9(c), Co doping results in the increase of the a.c. conductivity, especially at higher frequency, which is consistent with the results reported, as shown by Mehnaz *et al.*^52^ As has been established before, *α*_a.c._ gradually grows with higher frequencies at first, before increasing dramatically at high octaves. *α*_a.c._ observed behavior is consistent with the predictions of the Hopping Model. Since low-frequency transport occurs along an unlimited number of pathways, the *α*_a.c._ remains constant at such frequencies. However, when the frequency increases, so does the Hopping process, leading to a higher *α*_a.c._. Increased charge carrier mobility is achieved by charge carrier hopping between Zn^2+^, Co^2+^ and Yb^3+^ ions, which in turn increases the *α*_a.c._. The following equation describes the *α*_a.c._ of all samples of ZnO, that were doped at different Yb content.

4 *α*_a.c_ = *ε*_o_*ε*′′*ω*

As the eqn (3) suggests *α*_a.c._ is solely dependent on the dielectric loss. Since the *α*_a.c._ grows larger at higher frequencies, the dielectric loss lowers. The series resistance effect has been demonstrated in the literature to account for the linear frequency-dependent rise in a.c. intensity; therefore this finding is consistent with that finding.^53^ Indeed, the enhancement of a.c. could be due to either the high ac-frequency electric energy, which can facilitate the hopping of charge carriers between the nanoparticles, enhanced dielectric relaxation of the high-frequency polarization in nanoparticles of zinc oxide and nickel dioxide. As a result, it has been proposed so that the (Co, Yb) co-ZnO nanoparticles could serve as promising materials for use in advanced batteries. Increased electron transmission for uses such as gas sensing may also result from the larger *α*_a.c_.^53^

### 3.3 Magnetic properties

**Fig. 10:**
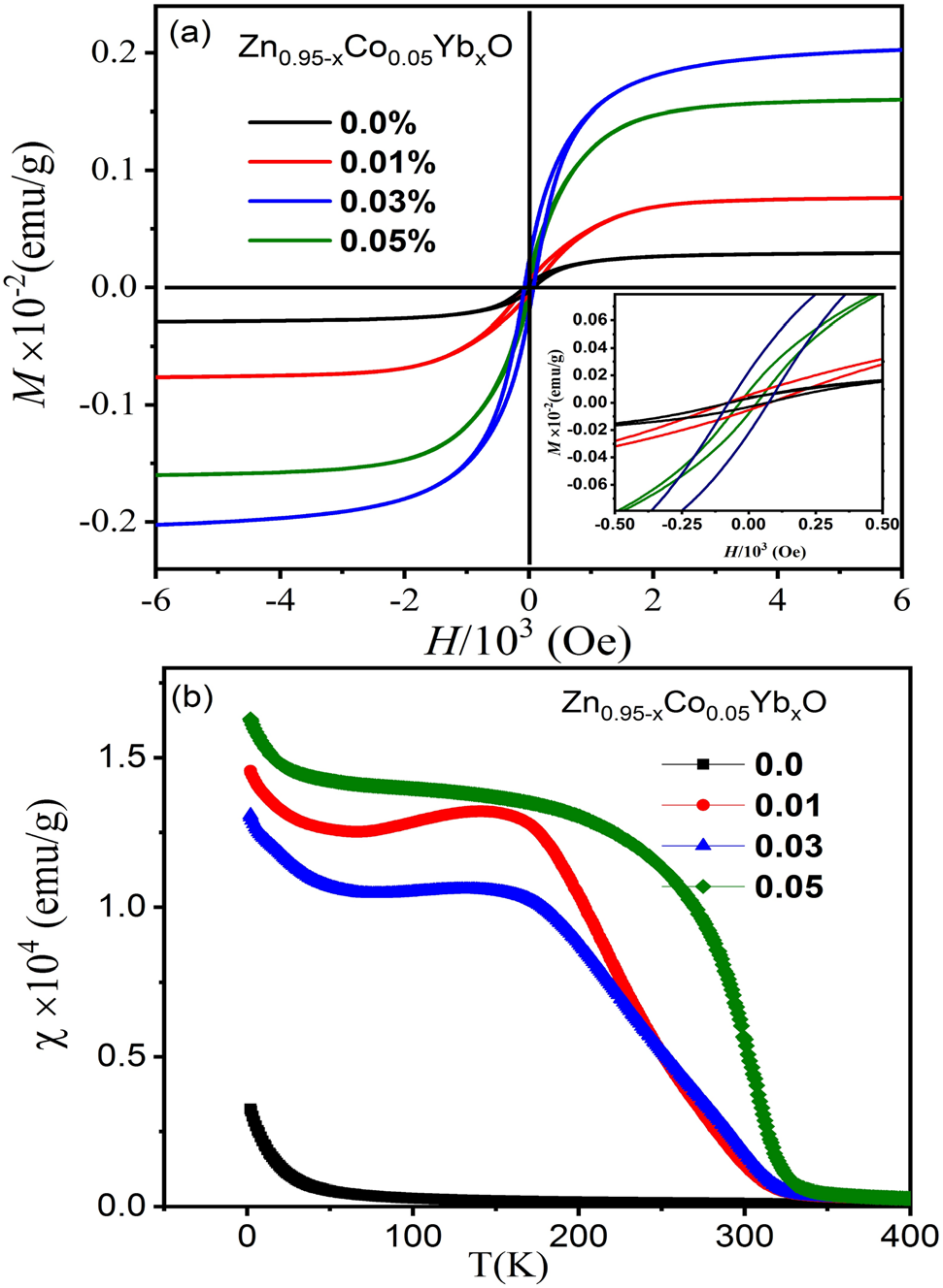
(a) The magnetic hysteresis (M–H) loops of the (Co, Yb) co-doped ZnO NPs and (b) their corresponding temperature-dependent magnetization.

Fig. 10(a) illustrates the magnetic hysteresis (M–H) curves for ZnO NPs co-doped with Co and Yb, measured at room temperature. In the case of pure ZnO, the M–H curve exhibits diamagnetism at 300 K, although its specific data is not presented here. However, for Zn_0.95_Co_0.05_O and Zn_0.95−*x*_Co_0.05_Yb_*x*_O samples (with Co 5% and varying Yb concentrations *i.e.*, 1, 3 and 5%), a clear ferromagnetic (FM) response is observed. In Fig. 10(a), we observe the remanent magnetization (Mr) values for the Zn_0.95−*x*_Co_0.05_Yb_*x*_O (*x* = 0.0.01, 0.03 and 0.05) samples with different Yb compositions: approximately 0.005 × 10^−3^ emu g^−1^, 0.012 × 10^−3^ emu g^−1^, 0.016 × 10^−3^ emu g^−1^, and 0.020 × 10^−3^ emu g^−1^, along with coercive field values of 57 Oe, 87 Oe, 159 Oe, and 252 Oe, respectively. Additionally, the saturation magnetization values are reported as 0.02 × 10^−2^, 0.05 × 10^−2^, 0.155 × 10^−2^, and 0.195 × 10^−2^ emu g^−1^ in Table 2. It is noteworthy that the *M*_r_ value for the Zn_0.92_Co_0.05_Yb_0.03_O specimen surpasses values reported in the literature.^33^ The transition from paramagnetic to ferromagnetic states is evident in Fig. 10(a). Notably, the *M*_r_ values of Zn_0.95−*x*_Co_0.05_Yb_*x*_O (Co = 0.03) specimens exceed those of Co–ZnO. This enhancement can be attributed to the effect of O^2^ annealing, which increases the doping levels of Co and Yb ions within the host lattice, consequently leading to a higher density of defects. The room-temperature ferromagnetism (RTFM) observed in the 3% Yb co-doped specimen may arise from both intrinsic and extrinsic magnetism sources. Extrinsic sources involve the formation of clusters of transition elements or secondary phases, while intrinsic sources pertain to exchange interactions within the material.

**Table 2:** The magnetic parameters of (Co, Yb) co-doped ZnO magnetic NPs

Samples	*M*_r_ (emu g^−1^)	*H*_c_ (Oe)	*M*_s_ (emu g^−1^)
Zn_0.95_Co_0.05_O	0.005 × 10^−3^	57 ± 0.04	0.020
Zn_0.94_Co_0.05_Yb_0.01_O	0.012 × 10^−3^	87 ± 0.09	0.05
Zn_0.92_Co_0.05_Yb_0.03_O	0.016 × 10^−3^	159 ± 0.05	0.155
Zn_0.90_Co_0.05_Yb_0.05_O	0.020 × 10^−3^	252 ± 0.04	0.198

To provide a more comprehensive understanding of the magnetic properties, temperature-dependent magnetization plots under a magnetic field of 10^3^ Oe are presented in Fig. 10(b). These data reveal that Zn_0.96−*x*_Yb_*x*_Co_*x*_O (Co = 0.03) samples exhibit a stronger ferromagnetic (FM) response compared to both pure Co-doped and highly Yb co-doped specimens. The underlying mechanisms of FM behavior in transition metal (TM)-doped ZnO materials have been previously elucidated in studies.^27^ It has been demonstrated that FM arises from the interaction between TM ions and bound polarons, leading to the formation of bound magnetic polarons. Furthermore, research on defect-bound charge carriers and hybridization of point defects has shed light on the generation of room-temperature ferromagnetism (RTFM) in TM-doped ZnO.

In this particular investigation, our findings indicate that O^2^ annealing plays a pivotal role in enhancing the incorporation of Co and Yb ions into the ZnO lattice. The resulting RTFM in Zn_0.95−*x*_Co_0.05_Yb_*x*_O nanotubes is attributed to the doping of Co^2+^ and Yb^3+^ ions, replacing Zn^2+^ and O^2^ in the lattice during the annealing process. This substitution creates O^2^ vacancies, crucial for maintaining charge balance within the material. This novel phenomenon emerges as a noteworthy outcome of our specimen preparation methodology. As depicted in Fig. 10(b), a noticeable decrease in the Curie temperature (*T*_C_), occurring at approximately 345 K, is observed in the *χ*(*T*) data for Zn_0.95−*x*_Co_0.05_Yb_*x*_O (*x* = 0.03). The observed reduction in magnetic moment with higher Yb concentrations may be linked to structural changes in the (Co, Yb) co-doped ZnO samples. These alterations in structural properties are correlated with variations in the unit cell parameters, particularly an increase in the lattice constants and consequently, the unit cell volume as the cobalt concentration increases. The expansion of the unit cell volume leads to a greater separation between neighboring cobalt ions within the ZnO matrix. This, in turn, results in antiferromagnetic super-exchange interactions between adjacent Co and Yb ions, thereby causing an increase in the magnetic moment. The impact of temperature on the inverse magnetic susceptibility of the nanoparticles is investigated in the temperature range from 2 K to 300 K. It is evident that the inverse susceptibility exhibits a linear decrease until reaching 345 K, at which point it deviates from the Curie–Weiss line. These findings provide insights into the exchange integral (*j*), which quantifies the interaction among magnetic ions within the material. Notably, the negative values of *θ* suggest that the magnetic dopants are engaged in relatively weak antiferromagnetic interactions.^50^ However, it is essential to acknowledge that the magnetic properties observed at low temperatures could potentially be influenced by manufacturing imperfections or the presence of impurity phases. A comprehensive understanding of this aspect necessitates further investigative research.

### 3.4 Memristive behavior of Au/(Co, Yb) co-ZnO/Au/Si–SiO_2_

**Fig. 11:**
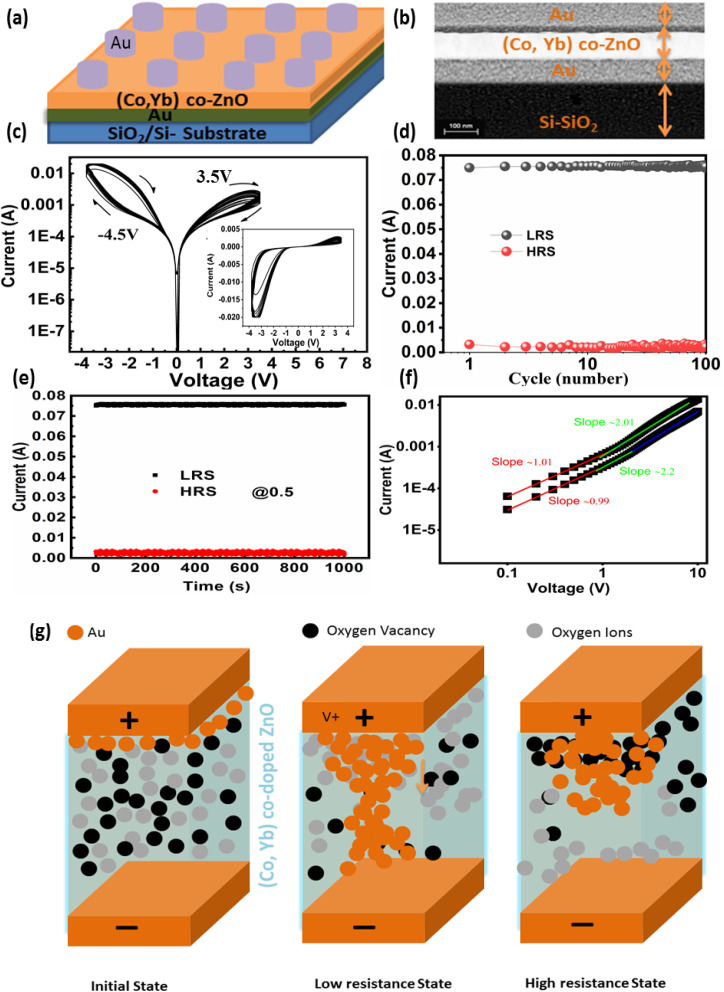
(a) show the Au/(Co, Yb) co-doped ZnO/Au/Si–SiO_2_ device schematic, (b) the TEM cross scan, (c) *I*–*V* characteristics of Au/(Co, Yb) co-doped ZnO/Au/Si–SiO_2_ (d) Endurance test performed 120 cycles in a sweep bias mood. The resistance values at HRS and LRS are taken at a read voltage of 1 V. (e) current retention time at HRS and LRS. The resistance values are measured while the voltage is held constantly at 0.5 V. (g) the switching mechanism can also be explained well by the fitted *I*–*V* curves of the positive region in a double logarithmic scale and (f) charge transport behavior of Au/(Co, Yb) co-doped ZnO/Au/Si–SiO_2_ device.

The schematic representation of the proposed sputter-grown memristor is illustrated in Fig. 11(a). This memristor configuration incorporates a central (Co, Yb) co-ZnO layer, situated between two electrodes composed of Au (both top and bottom electrodes). Further elaboration on the fabrication and characterization procedures of this device can be found in the Experimental section. To verify the structural integrity of the deposited layers in the Au/(Co,Yb) co-ZnO/Au/SiO_2_ sample, we conducted a cross-sectional analysis using TEM mapping, as depicted in Fig. 11(b). The TEM image, accompanied by a 100 nm scale bar, confirms the presence of a well-developed ZnO layer on the substrate, with a thickness of approximately 100 nm. Additionally, the thickness of both the top and bottom Au electrodes is roughly 100 nm. Our experimentation demonstrated repeatable analogue resistance switching, characterized by sequential increases and decreases in current and the presence of multiple resistance states over 120 DC switching cycles. This was achieved by gradually varying the voltage from +3.5 to −4.5 V, in steps of ±25 mV, as illustrated in Fig. 11(c). These observations clearly indicate the ability to manipulate the conductance of neuristors between low resistance states (LRS) and high resistance states (HRS) under varying voltage polarities. The statistical endurance properties of the LRS and HRS (read at 1 V) are presented in Fig. 11(d), revealing stable and reproducible resistive switching with minimal dispersion over the first 100 pulse switching cycles at room temperature. In Fig. 11(e), we examine the retention properties of the Au/(Co,Yb) co-ZnO/Au/SiO_2_ device, with different current states achieved by adjusting the SET stop voltages (0.5 V). Importantly, our memristor exhibits negligible degradation of the memory window at room temperature, as shown in Fig. 11(e), indicating excellent thermal stability. The formation of conductive filaments (CFs) in oxide-based RRAM devices plays a pivotal role during the switching process.^54^ This phenomenon is influenced not only by the choice of materials but also by chemical reactions within the switching layer, including ion migration, thermal chemical reactions, and electrochemical metallization. These chemical reactions predominantly occur in oxide-based RRAM devices with an active electrode, such as Au.^54^ To gain deeper insights into the switching mechanism, we propose a physical model for the Au/(Co,Yb) co-ZnO/Au/SiO_2_ device in Fig. 11(f), which depicts the initial HRS of the device without any external bias. During the “set” operation, when a positive voltage is applied to the top electrode (TE) Au, an electric field is generated between the TE and bottom electrode (BE), resulting in the generation of Au ions (Au+) within the (Co,Yb) co-ZnO layer. These Au ions migrate towards the BE through the SiO_2_ layer, where they accept electrons from the BE and are subsequently reduced to metallic Au atoms (Fig. 11(f)). The repeated redox reactions lead to the formation of a robust Au CF, ultimately bridging the two electrodes and thereby changing the resistance of the RRAM device from HRS to LRS (Fig. 11(f)). During the “reset” operation, when a negative voltage is applied to TE Ag, the opposite electric field and the induced Joule heating effect resulting from the repeated redox reactions disrupt the CF, allowing for the recovery of Au atoms from the BE.^55,56^ This action switches the RRAM device from LRS to HRS (Fig. 11(f)).

5 Au = Au^+^ + e^−^

6 Au^+^ + e^−^ = Au

Furthermore, the switching mechanism is aptly explained by the fitted *I*–*V* curves in the positive region, depicted in a double logarithmic scale (Fig. 11(g)). These curves indicate the dominance of the space-charge limited current (SCLC) conduction mode in Au/(Co,Yb) co-ZnO/Au/SiO_2_ RRAM devices. In the lower voltage region, the current exhibits a proportional relationship with voltage (*I* ∝ *V*, slope = 0.9), indicative of ohmic behavior. Conversely, in the higher voltage region, the current initially increases with a slope of 2.3, conforming to Child's square law and following the SCLC conduction mode.^57^ In the negative region, the current displays an increase with a slope of 1.1, indicating ohmic behavior once again. These findings underscore the crucial role of CF formation in RRAM devices utilizing stacked Au/(Co,Yb) co-ZnO/Au/SiO_2_ on SiO_2_ with Au TE. These results demonstrate the precise tuning of the total resistance in the testing device under a sweeping voltage mode, with analog switching characteristics that enable the device to function effectively as a biological synapse.

## 4. Summary

This manuscript explores the fascinating properties of (Co, Yb) co-doped zinc oxide (ZnO) nanoparticles with a particular focus on their potential application in neuromorphic computing. Through a combination of hydrothermal nanoparticle synthesis and target sputtering techniques, cobalt (Co) and ytterbium (Yb) were successfully introduced into the ZnO structure. This incorporation of dopants resulted in several noteworthy changes in the material's characteristics. One significant outcome of the doping process was the increase in oxygen vacancies and grain volume within the ZnO structure, indicating grain growth. This growth was accompanied by a reduction in grain boundaries, which in turn led to an enhancement in electrical conductivity. Most notably, the Co and Yb-doped ZnO nanoparticles exhibited room-temperature ferromagnetism, opening up possibilities for the integration of magnetic functionalities into neuromorphic computing systems. The study also introduced a sputter-grown memristor featuring a (Co, Yb) co-ZnO layer sandwiched between gold (Au) electrodes. Characterization of the memristor confirmed the presence of the ZnO layer and 100 nm-thick Au electrodes. The memristor's functionality was a key focus, as it exhibited repeatable analog resistance switching, allowing for precise manipulation of conductance between low and high resistance states. This property is particularly valuable for neuromorphic computing, where synapse-like behavior is essential. Furthermore, the manuscript presented robust evidence of the memristor's stability and endurance. Statistical tests revealed stable resistive switching with minimal dispersion over the course of 100 pulse cycles at room temperature. The retention properties were equally impressive, with the memristors maintaining four distinct current states for extended periods, up to 1000 seconds. This demonstrated exceptional thermal stability, a vital characteristic for reliable neuromorphic computing applications.

## Data availability

On reasonable request, the corresponding author will make available the datasets used and/or created during this investigation. The experimental work and language of the manuscript are also unique. There was no evidence of plagiarism in the submitted manuscript. If the reviewer insists on seeing the evidence, we would gladly deliver it to them in a plagiarized form.

## Author contributions

All the author equally contributed to this artcile.

## Conflicts of interest

The authors declare that they have no known competing financial interests or personal relationships that could have influenced the work reported in this paper.

## Supplementary Material
