# Expression of concern: Enhanced electrical and magnetic properties of (Co, Yb) co-doped ZnO memristor for neuromorphic computing

**DOI:** 10.1039/d4ra90092h

**Published:** 2024-09-04

**Authors:** Noureddine Elboughdiri, Shahid Iqbal, Sherzod Abdullaev, Mohammed Aljohani, Akif Safeen, Khaled Althubeiti, Rajwali Khan

**Affiliations:** a Chemical Engineering Department, College of Engineering, University of Ha'il P.O. Box 2440 Ha'il 81441 Saudi Arabia; b Chemical Engineering Process Department, National School of Engineers Gabes, University of Gabes Gabes 6029 Tunisia; c Department of Physics, University of Wisconsin La Crosse WI USA; d Engineering School, Central Asian University Tashkent Uzbekistan; e Scientific and Innovation Department, Tashkent State Pedagogical University Named After Nizami Tashkent Uzbekistan; f Department of Physics, University of Poonch Rawalakot Rawalakot 12350 Pakistan; g Department of Chemistry, College of Science, Taif University P.O. BOX. 110 21944 Taif Saudi Arabia; h Department of Physics, University of Lakki Marwat Lakki Marwat KP Pakistan rajwali@ulm.edu.pk khan_phy@foxmail.com; i Department of Physics, United Arab Emirates University United Arab Emirates

## Abstract

Expression of concern for ‘Enhanced electrical and magnetic properties of (Co, Yb) co-doped ZnO memristor for neuromorphic computing’ by Noureddine Elboughdiri *et al.*, *RSC Adv.*, 2023, **13**, 35993–36008, https://doi.org/10.1039/D3RA06853F.

*RSC Advances* is publishing this expression of concern in order to alert readers that concerns have been raised regarding the integrity of the data published in Fig. 4.

An independent expert has assessed Fig. 4 and is concerned the authors have not provided an accurate representation of the experiments that were conducted.

The Royal Society of Chemistry has asked the affiliated institution (University of Lakki Marwat, Pakistan) to investigate this matter and confirm the integrity and reliability of the data in Fig. 4 of the paper. An expression of concern will continue to be associated with this manuscript until we receive information from the institution on this matter.

Laura Fisher

21st August 2024

Executive Editor, *RSC Advances*

