# Correction and removal of expression of concern: Enhanced electrical and magnetic properties of (Co, Yb) co-doped ZnO memristor for neuromorphic computing

**DOI:** 10.1039/d5ra90029h

**Published:** 2025-03-24

**Authors:** Noureddine Elboughdiri, Shahid Iqbal, Sherzod Abdullaev, Mohammed Aljohani, Akif Safeen, Khaled Althubeiti, Rajwali Khan

**Affiliations:** a Chemical Engineering Department, College of Engineering, University of Ha'il P.O. Box 2440 Ha'il 81441 Saudi Arabia; b Chemical Engineering Process Department, National School of Engineers Gabes, University of Gabes Gabes 6029 Tunisia; c Department of Physics, University of Wisconsin La Crosse WI USA; d Engineering School, Central Asian University Tashkent Uzbekistan; e Scientific and Innovation Department, Tashkent State Pedagogical University Named After Nizami Tashkent Uzbekistan; f Department of Physics, University of Poonch Rawalakot Rawalakot 12350 Pakistan; g Department of Chemistry, College of Science, Taif University P.O. Box 110 21944 Taif Saudi Arabia; h Department of Physics, University of Lakki Marwat Lakki Marwat KP Pakistan rajwali@ulm.edu.pk khan_phy@foxmail.com; i Department of Physics, United Arab Emirates University United Arab Emirates

## Abstract

Correction and removal of expression of concern for ‘Enhanced electrical and magnetic properties of (Co, Yb) co-doped ZnO memristor for neuromorphic computing’ by Noureddine Elboughdiri *et al.*, *RSC Adv.*, 2023, **13**, 35993–36008, https://doi.org/10.1039/D3RA06853F.

The authors regret that due to an error in the data labels, the incorrect data was used for Fig. 4b.

The University of Lakki Marwat, Pakistan has investigated and confirmed the integrity and reliability of the EDX data associated with Fig. 4b and the new SEM data for Fig. 4, which are shown here.

**Fig. 4:**
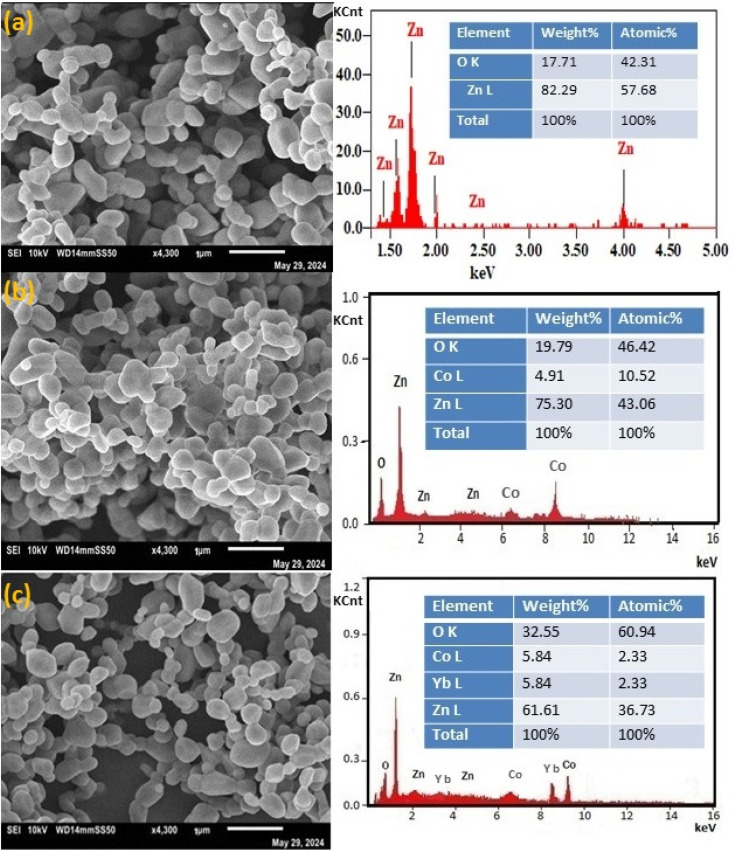
(a–c) shows the SEM images and their corresponding (right panel of Fig. 2) EDX of (a) ZnO, (b) Zn_0.95_Co_0.05_O and (c) Zn_0.92_Co_0.05_Yb_0.05_O NPs.

The University of Lakki Marwat, Pakistan investigation found that as there is no SEM instrument at the institution the SEM analysis was outsourced to a collaborating institution. During this process, a communication error occurred between the graduate author and the SEM operator who collected the data for Fig. 4. This error resulted in the same data mistakenly being used for Fig. 4a and b of the original publication. The investigating committee were able to confirm that this overlap was caused by miscommunication between the graduate author and the SEM operator and was not due to deliberate misconduct. The original data for Fig. 4a and c are correct. New SEM data was collected from a separate collaborating institution and the investigating committee was able to verify the integrity and reliability of the new data.

This correction supersedes the information provided in the Expression of concern related to this article.

The Royal Society of Chemistry apologises for these errors and any consequent inconvenience to authors and readers.

